# MCMV-infected maize attracts its insect vector *Frankliniella occidentalis* by inducing β-myrcene

**DOI:** 10.3389/fpls.2024.1404271

**Published:** 2024-08-21

**Authors:** Weiling Huang, Shujun Wei, Tao Zhou, Zaifeng Fan, Lijun Cao, Zhihong Li, Shaokun Guo

**Affiliations:** ^1^ Department of Plant Biosecurity, College of Plant Protection, China Agricultural University, Beijing, China; ^2^ Key Laboratory of Surveillance and Management for Plant Quarantine Pests of Ministry of Agriculture and Rural Affairs, Beijing, China; ^3^ Institute of Plant Protection, Beijing Academy of Agriculture and Forestry Sciences, Beijing, China

**Keywords:** plant volatiles, indirect virus-vector interaction, *Frankliniella occidentalis*, maize chlorotic mottle virus, multi-omics profiling, semi-persistent transmission, β-myrcene

## Abstract

Maize lethal necrosis is attributed to the accumulation of maize chlorotic mottle virus (MCMV), an invasive virus transmitted by insect vectors. The western flower thrips (WFT) can shift host to maize, thus promoting the spread of MCMV. However, our understanding of the characteristics and interactions involved in the transmission of MCMV is still limited. This study finds that non-viruliferous WFTs showed a 57.56% higher preference for MCMV-infected maize plants compared to healthy maize plants, while viruliferous WFTs showed a 53.70% higher preference for healthy maize plants compared to MCMV-infected maize plants. We also show for the first time that both adults and larvae of WFT could successfully acquire MCMV after 1 min of acquisition access period (AAP), and after 48 h of AAP, WFT could transmit MCMV in an inoculation access period of 1 h without a latent period. Both adults and larvae of WFT can transmit MCMV for up to 2 days. Furthermore, the decreasing number of viruliferous WFTs and transmission rates as time progressed, together with the transcriptomic evidence, collectively suggest that WFTs transmit MCMV in a semi-persistent method, a mode of transmission requiring minutes to several hours for acquisition access and having a retention time of several hours to a few days. Additionally, β-myrcene can attract WFTs significantly and is detected in *Nicotiana benthamiana* plants transiently expressing MCMV CP (coat protein), which is consistent with results in MCMV-infected maize plants through the metabolomic profiling and the preference analyses of WFT. Therefore, this study demonstrates the indirect interaction between MCMV and WFT by inducing maize to synthesize β-myrcene to attract insect vectors. The exploration of specific interactions between MCMV and WFT could help to expand the mechanism studies of virus–vector–host plant interaction and put forward a new insight for the combined control of MCMV and WFT through the manipulation of plant volatiles and key insect genes.

## 1 Introduction

Maize lethal necrosis (MLN), which results in leaf necrosis, premature aging, and even whole plant death, is a maize disease caused by synergistic infection with maize chlorotic mottle virus (MCMV) and one of several viruses from *Potyviridae* (Redinbaugh and Stewart, 2018; Jiao et al., 2022). This synergistic infection promotes MCMV accumulation, which plays a significant role in the development and perpetuation of MLN epidemics (Redinbaugh and Stewart, 2018). Maize-infecting potyviruses are distributed worldwide, but the emergence of MCMV is the main reason causing the outbreaks of MLN (Redinbaugh and Stewart, 2018). MCMV is the only member of the genus *Machlomovirus* in the family Tombusviridae (Jiao et al., 2021), whose hosts are limited to some monocotyledonous plants, including maize, sorghum, and sugarcane as the natural hosts, and barley, wheat, and millet as the experimental hosts (Huang et al., 2016). Over the past decade, MCMV has spread globally (Bernardo et al., 2023). MCMV was reported in Peru in 1974, Kansas in 1978, Texas in 1979, Argentina and Brazil in 1981, Thailand in 1982, and, subsequently, Mexico and Hawaii in 1990 and Asia and East Africa in 2011 (Castillo and Hebert, 1974; Wangai et al., 2012; Cabanas et al., 2013). In China, MCMV was listed in the catalogue of quarantine pests for import plants to China in 2007 and the national list of agricultural plant quarantine pests in 2020 (Ministry of Agriculture and Rural Affairs of the People's Republic of China, 2020; General Administration of Customs of the People's Republic of China, 2021). The distribution of MCMV has been reported in Yunnan province, and it is estimated that under the no-prevention scenario, 90% confidence interval of the total potential economic loss caused by MCMV to China’s maize industry was 32.944–50.896 billion RMB (Xie et al., 2011; Wei et al., 2022).

The genome of MCMV encodes seven proteins, including P7a, P7b, P31, P32, P50, P111, and CP (Redinbaugh and Stewart, 2018). Among them, CP as the structural protein is usually identified as viral determinants of insect transmission for virus (Ng and Falk, 2006; Hogenhout et al., 2008). Insect vectors provided an effective transmission mode for plant virus to expand the scope of damage in the field, and nearly 80% of plant virus are transmitted by insects (Hogenhout et al., 2008; Gilbertson et al., 2015; Lefeuvre et al., 2019). In the same way, MCMV could be transmitted by *Frankliniella williamsi*, *Frankliniella occidentalis* (western flower thrip, WFT) (*Thysanoptera*: *Thripidae*), and many beetles (Nault et al., 1978; Cabanas et al., 2013; Zhao et al., 2014).

Among them, WFT is an important invasive pest and supervector of plant viruses, which is characterized by a tiny size, wide range of hosts, high fecundity, and strong virus-transmission capability (Morse and Hoddle, 2006; Gilbertson et al., 2015). Therefore, WFT could not only damage plants but also affect yield and quality of important economic crops by causing significant plant virus diseases (Jones, 2005; Wu et al., 2018; Reitz et al., 2020). It is reported that WFT could transmit 31 viruses that belonged to at least five genera, including *Ilarvirus*, *Machlomovirus*, *Carmovirus*, *Sobemovirus*, and *Tospovirus* (Gilbertson et al., 2015; He et al., 2020). Previous studies have shown that WFT could transmit MCMV in a semi-persistent manner (Mwando et al., 2018). However, there is still a lack of strong evidence.

Insect vectors transmit plant viruses in two distinct ways, including circulative and non-circulative transmission. Circulative virus could be retained in large numbers and for a long time in the vectors, also known as persistent transmitted virus, which was verified to directly manipulate behaviors of insects by changing their immune system, nervous system, or endocrine system (Blanc and Michalakis, 2016; Oliver and Whitfield, 2016). The transmission mechanism by WFT of an important persistent transmitted virus, tomato spotted wilt virus (TSWV), was a hotspot in previous research (Whitfield et al., 2005; Bahat et al., 2020). For non-circulative viruses including non-persistent and semi-persistent transmitted virus, the acquisition and transmission processes of insect vectors are completed in a relatively short time without a latent period. Thus, frequent piercing, feeding sites changing, and host transfer are more beneficial and efficient for the transmission of non-circulative viruses (Ng and Zhou, 2015; Blanc and Michalakis, 2016; Zhou et al., 2018).

There are many well-studied plant viruses transmitted by insect vectors in a semi-persistent manner, such as lettuce infectious yellows virus transmitted by *B. tabaci* (Duffus et al., 1986; Ng and Falk, 2006; Chen et al., 2011), cauliflower mosaic virus transmitted by aphids (Ng and Falk, 2006; Uzest et al., 2010; Hohn, 2013), and wheat streak mosaic virus transmitted by mites (Bragard et al., 2013). The above semi-persistent viruses could not replicate inside insect vectors, but indirectly manipulate insect vectors’ behavior through the utilization of volatiles from host plants to achieve efficient dispersal (Mauck, 2016; Mwando et al., 2018; Hammerbacher et al., 2019). It is reported that MCMV could induce changes in the volatiles of host plants, attracting thrips vectors and enhancing virus transmission (Mwando et al., 2018). WFT is a polyphagous pest that can feed on over 500 species of crops from more than 60 families (Reitz et al., 2020; Jin et al., 2023), including *Asteraceae*, *Oleaceae*, *Solanaceae*, *Lamiaceae*, *Fabaceae*, and *Poaceae* in both greenhouses and open fields (Atakan, 2019; Uzun et al., 2022). It is a major horticultural pest worldwide, and vegetables and flowers are its main hosts, while maize is not (Zhang et al., 2019a; Hu et al., 2021). However, there is a strong evidence that WFT could transmit MCMV from maize (Zhao et al., 2014; Redinbaugh and Stewart, 2018), and it is not clear how MCMV affects WFT to facilitate its own spread and whether the release of volatiles from MCMV-infected maize induced WFT feeding.

In this study, we first verified the semi-persistent transmission characteristics of MCMV in larvae and adults through bioassay and transcriptomic analyses. Second, we detected the selection preferences of non-viruliferous and viruliferous WFTs to MCMV-infected and healthy maize plants. Third, from the metabolomic profiling of MCMV-infected maize plants, we selected the significant volatiles and verified the behavioral tendency of WFTs through Y-tube olfactometer assay. Finally, we verified the induction of significant volatiles by MCMV in *Nicotiana benthamiana* plants. These results could not only reveal the molecular mechanism of interaction between MCMV and WFT but also provide new insights for the development of quarantine prevention and control of these two pests.

## 2 Materials and methods

### 2.1 WFT, virus, and plants

WFTs were reared on kidney bean (*Phaseolus vulgaris*) in cylindrical tubes with two ends open (outer diameter 10 cm × height 26 cm) at 26°C, 60 ± 5% relative humidity (RH), and a photoperiod of 14: 10 (L: D) in the laboratory to simulate natural environmental conditions observed in maize-growing regions to provide the right feed, photoperiod, temperature, and humidity as previously described (Zhang et al., 2019b). MCMV was provided by Plant Virus Team from China Agricultural University. Inoculations of MCMV were performed as described in a previous study (Jiao et al., 2021). Briefly, crude extracts were prepared by homogenizing the MCMV-infected maize leaves in 0.01 M phosphate buffer, and each maize seedling was inoculated on the second leaf (i.e., the first true leaf) with 10 μL crude extracts. The plants of *N. benthamiana* and maize inbred line B73 were grown in a controlled greenhouse at 25 ± 1°C with a 70% ± 10% RH and a photoperiod of 14:10 (L:D).

### 2.2 Detection of MCMV in WFT

The MCMV in WFT was detected using a RT-qPCR (reverse transcriptase quantitative PCR) method. Total RNA of an individual WFT from infected maize plants were extracted using Micro-RNA Extraction Kit (Tianmo, Beijing, China) following the manufacturer’s instructions. Total RNA (50 ng) from each sample was reverse transcribed to synthesize the first-strand cDNA using the PrimeScript™ RT reagent Kit with gDNA Eraser (Perfect Real Time) (Takara, Kyoto, Japan). RT-qPCR was performed on a QuantStudio 6 system using TB Green^®^ Premix Ex Taq™ II (Tli RNaseH Plus) (Takara, Kyoto, Japan). Gene-specific primers for RT-qPCR were designed based on the MCMV *CP* gene sequence (**Supplementary Table S1**) and synthesized by Tsingke Biotech Co., Ltd. (Beijing, China). All reactions were carried out in a final volume of 25 μL containing the following: 12.5 μL of TB Green Premix Ex Taq™ II, 9 μL of RNase free dH_2_O, 0.5 μL of ROX, 1 μL of cDNA, and 1 μL of each primer. Amplification reactions were performed as follows: 95°C for 30 s and 40 cycles of 95°C for 5 s, and 60°C for 34 s. The housekeeping gene, *actin*, was used as internal reference genes for WFT (Rotenberg et al., 2009). Three independent biological replicates were performed, and relative gene expression was calculated using the 2^−ΔΔCt^ method (Livak and Schmittgen, 2001).

### 2.3 Transmission bioassay

#### 2.3.1 Acquisition access period (AAP)

A total of 10 WFTs from each MCMV-infected maize seedling were collected at time intervals following 1 min, 10 min, 0.5 h, 12 h, 24 h, and 48 h. WFTs feeding on healthy maize were served as a negative control. The total RNA of an individual WFT was extracted and detected by RT-qPCR as introduced above.

#### 2.3.2 Retention time

WFTs were released on two MCMV-infected maize plants covered by insect-proof cage. Two days after feeding on the MCMV-infected maize plants, 200 living WFTs were collected and starved for 2 h. Subsequently, they were placed into Petri dishes containing kidney beans, which are the non-host of MCMV, for feeding. The kidney beans were replaced every 3 days to remove newly hatched WFTs. A total of 10 WFTs were collected at time intervals of 12 h, 2 days, 4 days, 6 days, 8 days, 10 days, 18 days, 21 days, and 28 days after feeding. WFTs feeding on healthy maize plants for 2 days were served as a negative control. The total RNA of an individual WFT was also extracted and detected as described above.

#### 2.3.3 Inoculation access period

After 48 h of acquisition access period (AAP) and 2 h of starvation, twenty WFTs were placed on healthy maize leaves individually with clip cage for inoculation access period (IAP) of 1 h, 3 h, 6 h, 12 h, 24 h, and 48 h, respectively. Maize plants feeding by non-viruliferous WFTs were served as a negative control. Seven days after removing thrips, total RNA of individual leaf fed by thrips was extracted using TRIzol^®^ Reagent (Invitrogen, Carlsbad, CA, USA), and the first-strand cDNA synthesis and RT-qPCR were performed as described above. Gene-specific primers for *CP* were the same as mentioned above and the house keeping gene, *folypolyglutamate synthase* (*FPGS*), was used as internal reference genes for maize (Hurni et al., 2015) (**Supplementary Table S1**). Three independent biological replicates were performed, and relative gene expression was calculated using the 2^−ΔΔCT^ method as mentioned above.

We also used leaf disks assay to determine the transmission capacity by WFT. After 48 h of AAP and 2 h of starvation, WFTs were transferred to leaf disk chambers containing healthy maize leaves. The chambers were sealed with stretched parafilm, perforated at the top, and covered with the nylon mesh for ventilation. The maize leaves in the chambers were replaced daily, and the removed leaves were incubated at 25 ± 2°C for another 2 days to promote virus replication. Subsequently, the presence of MCMV was tested for up to 12 days using RT-qPCR as mentioned above. Maize leaves fed by non-viruliferous WFTs were served as a negative control, and five independent biological replicates, with each replicate consisting of 15 adults or larvae, were performed.

### 2.4 Selection preferences of WFT to MCMV-infected maize plants

**Figure 1:**
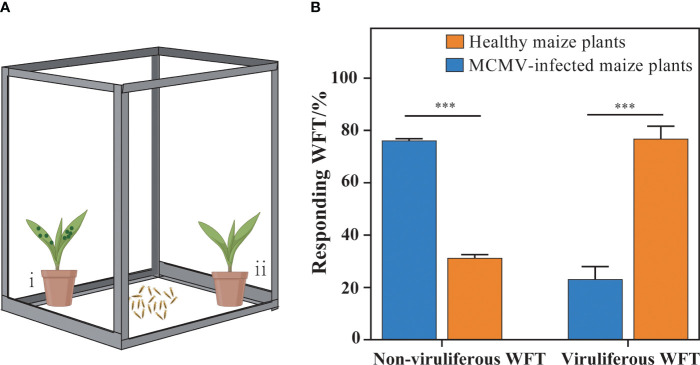
Selection preferences of western flower thrips (WFTs) to MCMV-infected plants. **(A)** The illustration of the experimental design for preference behaviors of WFTs to healthy and MCMV-infected maize plants. In an isolated cage (length 28 cm × width 38 cm × height 53 cm), **(i)** MCMV-infected maize plants were placed at one corner, **(ii)** while healthy plants were placed in the other corner. **(B)** The comparison of the responding WFT attracted to healthy and MCMV-infected maize plants. The symbols “***” above the bars represent significant differences at *p* < 0.001.

To investigate whether MCMV contributed to attracting WFTs to maize plants, we designed a selection experiment to analyze the preference behaviors of WFTs for MCMV-infected maize plants and healthy plants (**Figure 1A**). The MCMV-infected and healthy maize plants were placed at two opposite corners in an isolated cage to test the behavioral preference of WFTs. Non-viruliferous and viruliferous WFTs were collected and starved for 2 h before putting into the center of the cage, respectively. Six biological replicates, each containing 10 individuals, were set up for each treatment. To eliminate the influence of environmental variables, the positions of the MCMV-infected maize plant and the healthy maize plant were alternated after each repetition. The numbers of WFTs attracted to MCMV-infected and healthy maize plants were counted after 10 min.

### 2.5 Selection preferences of WFTs to *N. benthamiana* transiently expressing MCMV P32 and CP

We transiently expressed the key proteins of MCMV in *N. benthamiana* leaves to investigate their impact on the preference behaviors of WFTs. The utility of a pGD vector following agroinfiltration into leaves has been demonstrated with CP and P32 from MCMV (Goodin et al., 2002). The *N. benthamiana* leaves transiently expressed P32, CP, and GFP (green fluorescent protein), and a blank control was placed at four corners in an isolated cage to test the behavioral responses of WFTs. Non-viruliferous WFTs were collected and starved for 2 h before putting into the center of the cage. Three biological replicates, each containing 40 individuals, were set up for each treatment. The numbers of WFTs attracted to *N. benthamiana* with different treatments were counted after 0.5 h, 1 h, 2 h, 5 h, and 8 h.

### 2.6 RNA-seq and analyses

Adults and larvae of MCMV-viruliferous and non-viruliferous WFTs were collected at the same time of 1 day for transcriptome sequencing and gene expression analysis. A total of 200 WFTs were brushed onto the MCMV-infected maize leaves, while another 200 WFTs were applied to the healthy maize leaves for 48 h to obtain viruliferous and non-viruliferous WFTs. Total RNA was extracted using TRIzol^®^ Reagent (Invitrogen, Carlsbad, CA, USA) following the manufacturer’s instructions. Three biological replicates, each containing 50 individuals, were set up for each developmental stage. The quality and integrity of total RNA was verified using an UV-Vis Q5000 spectrophotometer (Quawell Technology Inc., San Jose, CA, USA) and agarose gel electrophoresis, respectively. TruSeq™ RNA sample preparation Kit from Illumina (San Diego, CA, USA) was used for cDNA libraries construction using 2 μg of total RNA. Paired-end library was sequenced with the Illumina NovaSeq 6000 sequencer (Illumina, San Diego, CA, USA) at the Majorbio Bio-pharm Technology Co., Ltd (Shanghai, China) after quality control. The analyses were performed with the online platform of the Majorbio I-Sanger Cloud Platform (www.i-sanger.com). We applied the DESeq2 to detect differentially expressed genes (DEGs) and adopted the well-established Benjamini–Hochberg method to calibrate *p-*values from the original assumption test (Ferreira and Zwinderman, 2006; Love et al., 2014). After calibration, the *p*-value was determined using the false discovery rate (FDR) approach to decrease false positives caused by independent statistical hypothesis testing on expression changes in a large number of genes. Candidate DEGs encoding putative chemosensory genes, immune response genes, and nutritional metabolism genes were identified from the KEGG and GO annotations (Conesa et al., 2005; Mao et al., 2005).

### 2.7 Metabolomic profiling

The samples of maize leaves from MCMV-infected and healthy maize plants were collected and treated with liquid nitrogen. Six biological replicates, each containing 0.6 g, were performed for each treatment. Metabolomic analyses were performed using gas chromatography–mass spectrometry (GC-MS) as described as previously (Guo et al., 2021; Yuan et al., 2022). Totally, 500 mg of the sample powder was transferred immediately to a 20-mL head-space vial (Agilent, Palo Alto, CA, USA), containing 1 mL of saturated NaCl solution and 10 μL (50 μg/mL) of internal standard solution. The volatiles were collected with a solid-phase microextraction (SPME, CTC Analysis AG, Switzerland). Chemical analysis was performed by GC-MS (8890-7000D) coupled with a 30 m × 0.25 mm × 0.25 μm DB5-MS (5% phenyl-polymethylsiloxane) capillary column (Agilent Technologies Inc., CA, USA). The confidence level is 70% and above. Selected ion monitoring (SIM) mode was used for the identification and quantification of analytes. For qualitative analysis, metabolites were compared to standards in the self-compiled database (MetWare, Wuhan, China) based on retention time, fragmentation pattern, and exact m/z values for accurate identification. For quantitative analysis, the signal intensity of metabolites was derived from distinctive ions. Integration and correction of selected quantitative ions enhance quantitative accuracy. The area under each chromatographic peak was indicative of the relative content of the corresponding substance. The internal standard was used to normalize the quantitative data. The fold change for each volatile was calculated in the comparison of MCMV-infected and healthy maize plants. The differential volatiles were selected based on a combination of a statistically significant threshold, including the variable influence on projection (VIP) values obtained from the Orthogonal Partial Least Squares Discriminant Analysis (OPLS-DA) model (VIP > 1) and absolute Log_2_FC (|Log_2_FC| ≥ 1.0). Identified volatiles were annotated using KEGG compound database (http://www.kegg.jp/kegg/compound/); annotated volatiles were then mapped to KEGG pathway database (http://www.kegg.jp/kegg/pathway.html). Pathways with significantly regulated volatiles were then fed into MSEA (volatile sets enrichment analysis); their significance was determined by hypergeometric test’s *p*-values.

### 2.8 Y-tube olfactometer assays

Behavioral responses of WFT adults to MCMV-induced maize volatiles were evaluated using a Y-tube olfactometer (Dicke et al., 1990; Ren et al., 2020). All adults were starved for 2 h before the experiments. The end of the two arms of the Y-tube olfactometer (diameter of 2.5 cm, base tube of 10 cm, arms of 10 cm, and arms angle of 45°) was connected to the odor source, containing a piece of filter paper (20 × 50 mm) loaded with 10 μL of different concentrations of 10^−3^ μg/μL, 10^−2^ μg/μL, 10^−1^ μg/μL, 1 μg/μL, and 10 μg/μL solution of six significant differential volatiles from the MCMV-infected maize plants and 10 μL of hexane as a blank control. The airflow was purified by activated charcoal and then passed through a gas-washing bottle with distilled water. Two streams of charcoal-purified air were each pumped into the olfactometer at a flowrate of 300 mL/min (Ren et al., 2020). All bioassays were carried out during daytime between 9:00 and 14:00 at a temperature of 25 ± 1°C and humidity of 40%. Five WFT adults were released at the stem of the Y-tube olfactometer and observed for 3 min. During the experiment, an individual moved into one-third of the length of one arm, and remaining there for at least 30 s was classified as “making a choice”. WFTs that did not enter either arm during 3 min were recorded as “no choice”. A total of 10 biological replicates were performed in the experiments, and the arms of the Y-tube olfactometer were reversed every five replications to avoid the potential side bias. To ensure the accuracy of the experiment, the Y-tube olfactometer was washed with ethanol and then heated at 180°C for 3 h before experiments and replaced after testing one concentration in the process of the experiment.

### 2.9 GC-MS conditions for verification of β-myrcene induction in *N. benthamiana*

The samples of *N. benthamiana* plants transiently expressing MCMV CP and blank control were collected. Two *N. benthamiana* plants were transferred immediately to a tightly sealed plastic box. The volatiles were collected with SPME for 1.5 h at room temperature. Volatile analysis was performed by GC-MS (7890A-5975C) (Agilent Technologies Inc., CA, USA). For SPME fiber analysis, samples were injected with splitless mode for volatile analysis at an initial oven temperature of 40°C (3.5 min hold), followed by a three-step temperature increase: first to 100°C (at a rate of 10°C/min), then to 180°C (at a rate of 7°C/min), and finally to 250°C (at a rate of 25°C/min, 5 min hold). Other parameters were injector temperature of 240°C, MS interface temperature of 240°C, and carrier gas (helium) at a flowrate of 1.2 mL/min.

### 2.10 Data analysis

SPSS 22.0 (SPSS, Inc., Chicago, IL, USA) and Graph Pad PRISM 9.0 (Graph Pad, Inc., San Diego, CA, USA) were used for statistical analyses and graphics, respectively. Heatmap packages (v1.0.12) of R language (v4.0.2) was used to draw heat maps (Chen et al., 2024). The statistical analysis of Y-tube bioassay and behavioral preferences of WFTs was determined by Student’s *t*-test (Wang et al., 2022; Chen et al., 2023). Asterisks denoted statistical significance between two groups (* *p* < 0.05, ** *p* < 0.01, and *** *p* < 0.001).

## 3 Results

### 3.1 Attraction of MCMV-infected maize to WFT and potential volatiles

The results show that 78.78% of the non-viruliferous WFTs are attracted to MCMV-infected maize plants, and 76.85% of the viruliferous WFTs are attracted to healthy maize plants (**Figures 1A, B**). Therefore, MCMV-infected plants attract non-viruliferous WFTs significantly, while viruliferous WFTs prefer the healthy maize plants.

### 3.2 Semi-persistent transmission of MCMV by WFT

**Figure 2:**
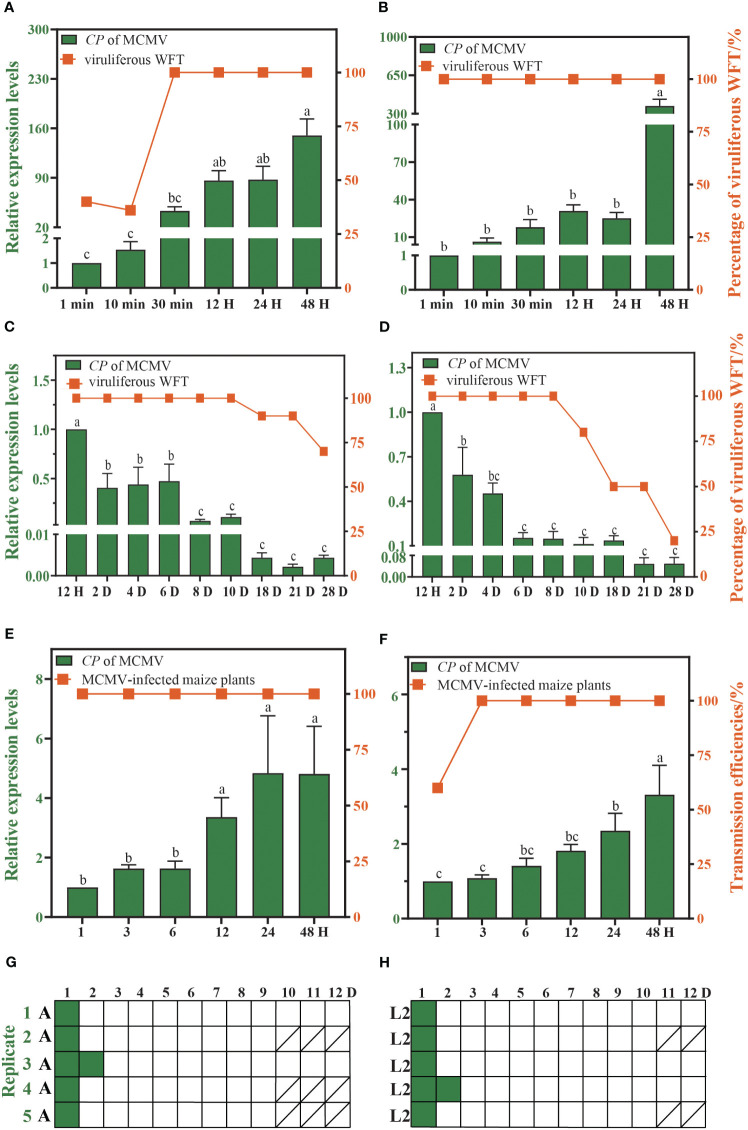
Characteristics of maize chlorotic mottle virus (MCMV) transmitted by western flower thrip (WFT). The acquisition access period (AAP) of WFT **(A)** adults and **(B)** larvae. The retention time of MCMV in WFT **(C)** adults and **(D)** larvae. The inoculation access period (IAP) of WFT **(E)** adults and **(F)** larvae. Pattern of MCMV transmission by WFT **(G)** adults and **(H)** larvae serially transferred to leaf disks (represented as boxes) daily. Green and white boxes represent leaf disks that were assayed to be positive and negative for MCMV by RT-qPCR, respectively. Bars show the standard error, and the different letters above the bars indicate significant differences at *p* < 0.05 as determined by one-way ANOVA. A, adults of WFT. L2, the second instar larvae of WFT./, all WFTs in this replicate died, and no maize leaves were collected.

The proportion of viruliferous WFTs increase with the duration of feeding on the MCMV-infected plants. WFTs need only 1 min of AAP to acquire MCMV successfully, and the proportion of viruliferous adults and larvae of WFT reaches 100% after 30 min and 1 min of AAP, respectively (**Figures 2A, B**). After 48 h of AAP, adults and larvae of WFT can retain MCMV for up to 28 days, and the proportion of viruliferous adults and larvae of WFT drops to 70% and 20% on the 28th day (**Figures 2C, D**). Within 1 h of IAP, WFTs can transmit MCMV, and the transmission efficiencies of adults and larvae are 100% and 60%, respectively (**Figures 2E, F**). After 48 h of AAP, adults and larvae of WFT could transmit MCMV for up to 2 days (**Figures 2G, H**, **Supplementary Table S2**).

### 3.3 Identification of DEGs between viruliferous and non-viruliferous WFTs

**Figure 3:**
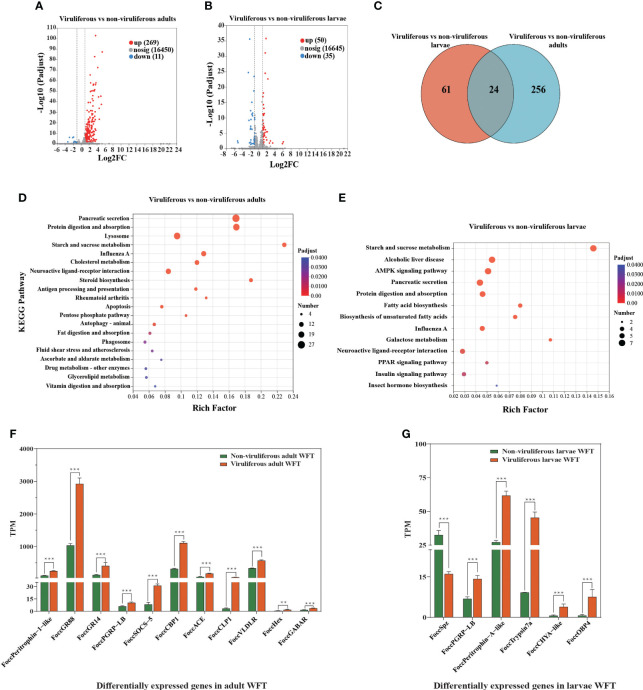
Differentially expressed genes (DEGs) from transcriptomic comparison between viruliferous and non-viruliferous western flower thrips (WFTs). Volcano plot of DEGs in **(A)** viruliferous adults and **(B)** viruliferous larvae. **(C)** Venn diagram showing the number of DEGs. KEGG pathways of DEGs in viruliferous **(D)** adults and **(E)** larvae. The bar plots of DEGs related to chemosensory, immune response, nutritional metabolism, and tubulin monoglutamylase in viruliferous **(F)** adults and **(G)** larvae. ***p < 0.001. **p <0.01.

After performing quality control, we obtain approximately 94.51 Gb of clean sequence data with Q30 values ≥ 95.40% (**Supplementary Table S3**). The percentage of each sample mapped to the reference genome ranges from 84.47% to 86.84%, indicating the reliability of the transcriptome data for subsequent analysis (**Supplementary Table S4**). Between MCMV-viruliferous and non-viruliferous WFTs, a total of 280 DEGs including 269 upregulated and 11 downregulated genes are identified in the adult stage, and a total of 85 DEGs including 50 upregulated and 35 downregulated genes are identified in the larvae stage (**Figures 3A, B**). The DEGs partially overlap in the adults and larvae stage, but most of them are specific in viruliferous adults (**Figure 3C**).

Adults of WFT exhibit an enrichment of KEGG pathways related to influenza A, apoptosis, autophagy, antigen processing, and presentation, while larvae related to influenza A, neuroactive ligand–receptor interaction, and pancreatic secretion (**Figures 3D, E**). We analyze the genes associated with immune response genes, nutritional metabolism genes, chemosensory-related genes, and chitin-binding genes. Among them, the nutritional metabolism genes (*Hexamerin*, *Hex*), the gustatory receptor (*GR*) including *GR14* and *GR88*, immune response genes including *cathepsin L-like peptidase* (*CLP*), *cathepsin B-like peptidase* (*CBP*), and *peptidoglycan-recognition protein LB-like* (*PRGP-LB*) are upregulated significantly. In addition, both of γ-aminobutyric acid receptor (GABAR) as the neurotransmitter receptors that regulate insect behavior and *peritrophin-1-like* as the chitin-binding gene are upregulated in the MCMV-viruliferous adults of WFT (**Figure 3F**, **Supplementary Tables S5, S6**). In the MCMV-viruliferous larvae, *OBP4*, *Trypsin*, *PGRP-LB*, *chymotrypsinogen A-like* (*CHYA-like*), and *peritrophin-A-like* are upregulated, while *Spz* (*Spatzle*) is downregulated (**Figure 3G**, **Supplementary Tables S5, S6**).

### 3.4 Volatiles of MCMV-infected maize

**Figure 4:**
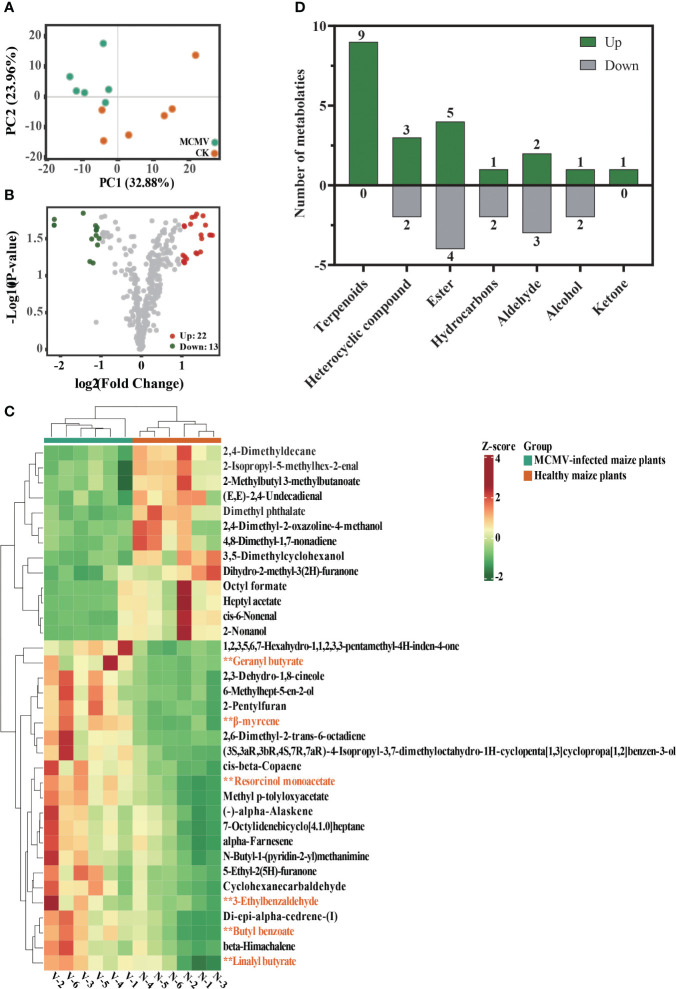
Metabolomic comparison between MCMV-infected and healthy maize plants. **(A)** Principal component analysis of all volatiles detected in maize. Data dots of different colors represent the different samples in the three groups. **(B)** Volcano plot of differential volatiles in MCMV-infected and healthy maize plants. **(C)** Heatmaps of differential volatiles in MCMV-infected and healthy maize plants. Asterisk represents six upregulated metabolic volatiles selected for subsequent Y-tube olfactometer assays. **(D)** Number of differential volatiles that were up- and downregulated in each of the different volatile classifications. N, healthy maize plants; V, MCMV-infected maize plants.

Principal component analysis (PCA) of all detected volatiles from metabolomic analysis indicates that the first two principal components (PCs) explain 56.84% of the total variation (**Figure 4A**). The PCA model generated for the two treatments demonstrates distinctive clustering patterns in the maize metabolome. The volcano plot and the heatmap show 22 upregulated and 13 downregulated volatiles in MCMV-infected maize plants (**Figures 4B, C**) (**Supplementary Table S7**). The identified volatiles are categorized into different classifications, with five downregulated and seven upregulated classes (**Figure 4D**). Among the 22 upregulated volatiles, nine are terpenoids and one is ketone that are absent in the downregulated volatiles. Among them, nine terpenoids constitute the highest number of volatiles.

### 3.5 Attraction of the upregulated volatile β-myrcene to WFTs

**Figure 5:**
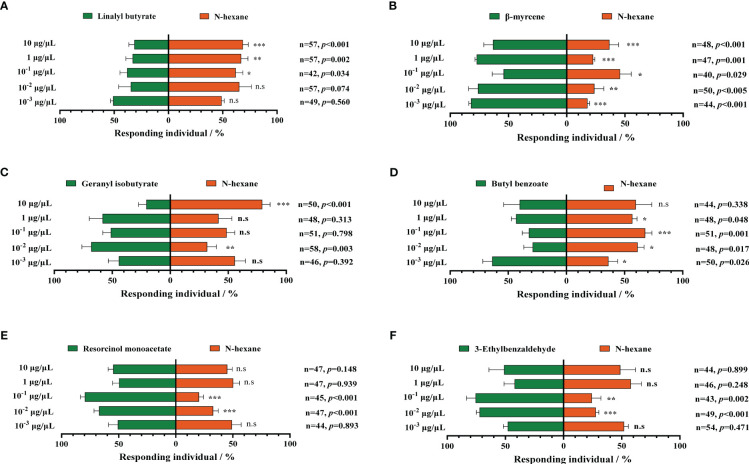
Behavioral responses of western flower thrips (WFTs) to six selected volatiles. At five concentrations ranging from 10^−3^ μg/μL to 10 μg/μL, the selection preference of WFTs to **(A)** linalyl butyrate, **(B)** β-myrcene, **(C)** geranyl isobutyrate, **(D)** butyl benzoate, **(E)** resorcinol monoacetate, and **(F)** 3-ethylbenzaldehyde. Asterisk indicates a significant difference within the dual-choice selection (***p < 0.001, **p < 0.01, *p < 0.05), and the abbreviation n.s indicates no significant difference (*p* > 0.05) (Student’s *t*-test).

We select six upregulated metabolic volatiles, including geranyl butyrate, butyl benzoate, resorcinol monoacetate, 3-ethylbenzaldehyde, linalyl butyrate, and β-myrcene from the metabolomic profiling of MCMV-infected and healthy maize, for verification of the attraction to WFTs. At the two concentrations of 10^−3^ μg/μL and 10^−2^ μg/μL, WFTs show no preference for linalyl butyrate and are avoided at the higher three concentrations (**Figure 5A**). Except for linalyl butyrate, WFT adults are attracted to the other five volatiles to some extent. Among them, only β-myrcene significantly attracts WFTs at five concentrations (**Figure 5B**). In addition, WFTs are attracted to geranyl isobutyrate at the concentration of 10^−2^ μg/μL (**Figure 5C**), to butyl benzoate at the concentration of 10^-3^ μg/μL (**Figure 5D**), to resorcinol monoacetate at the concentration of 10^−2^ μg/μL and 10^−1^ μg/μL (**Figure 5E**), to 3-ethylbenzaldehyde at the concentration of 10^−2^ μg/μL and 10^−1^ μg/μL (**Figure 5F**).

### 3.6 Validation of β-myrcene and attraction in *N. benthamiana* plants

**Figure 6:**
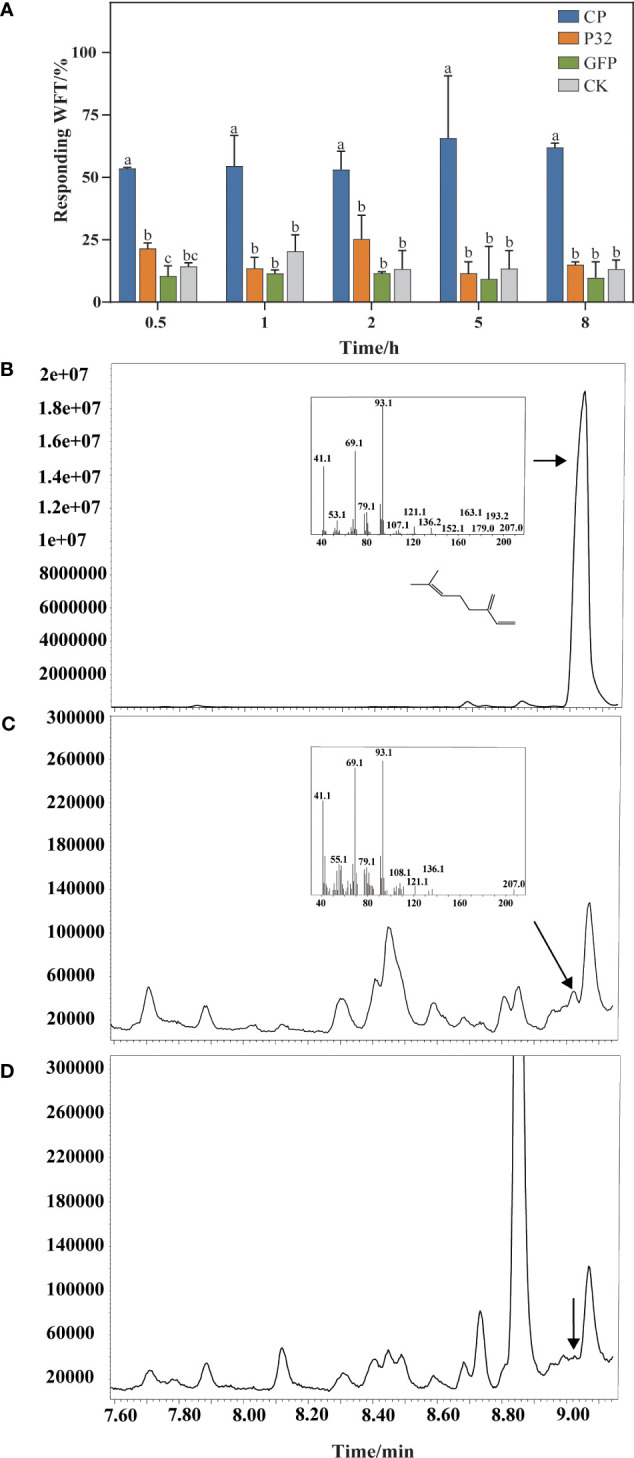
Validation of β-myrcene and attraction in *Nicotiana benthamiana* plants. **(A)** A comparison of western flower thrips (WFTs) attracted to *Nicotiana benthamiana* plants transiently expressing MCMV coat protein (CP), P32, GFP, and a blank control was conducted. **(B)** Standard of β-myrcene. **(C)** The products generated by *Nicotiana benthamiana* plants transiently expressed coat protein (CP) of MCMV. **(D)** The products generated by *Nicotiana benthamiana* without treatment (a blank control). β-Myrcene was identified by GC-MS according to the MS-library database. Different letters above the bars indicate significant differences at *p* < 0.05 as determined by one-way ANOVA.

At 0.5 h, the proportions of WFTs feeding on *N*. *benthamiana* leaves transiently expressed CP, P32, GFP, and plants as a blank control are 53.59%, 21.54%, 10.52%, and 14.36%, respectively. At 8 h, 61.94%, 15.04%, 9.78%, and 13.25% of WFTs tend to feed on *N. benthamiana* leaves transiently expressed CP, P32, GFP, and a blank control, respectively (**Figure 6A**). WFTs show a preference for the *N. benthamiana* leaves transiently expressed CP compared to P32, GFP, and blank control, and the preference trend is consistent at 0.5 h, 1 h, 2 h, 5 h, and 8 h. Through GC-MS, we verify β-myrcene in *N. benthamiana* plants transiently expressing CP when compared with β-myrcene standard in a retention time of 9.024 min (**Figures 6B, C**). However, β-myrcene is not detected in the blank control group (**Figure 6D**).

## 4 Discussion

In our study, an individual adult and larva of WFT required only 1 min of AAP to acquire detectable MCMV, and after 48 h of AAP, WFT could transmit MCMV within the first hour of IAP without a latent time. Both larvae and adults retained MCMV for 28 days with decreasing virus titer as time progressed but could transmit MCMV for up to 2 days after 48 h of AAP. Taken altogether, the results suggested that WFT transmitted MCMV in a semi-persistent manner. Our results are consistent with the MCMV transmission characteristics by *F. williamsi*, which has reported that both larvae and adults of *F. williamsi* transmitted MCMV after an AAP and IAP of 3 h (Cabanas et al., 2013). Whitefly and aphids can also acquire plant viruses in a relatively short AAP, such as 1 h of AAP for lettuce chlorosis virus (LCV) (Duffus et al., 1986), 2 h for cucurbit yellow stunting disorder virus (CYSDV) (Gil-Salas et al., 2007), 1 h for cucurbit chlorotic yellows virus (CCYV) (Li et al., 2019), and only 10 min for lettuce infectious yellows virus (LIYV) (Duffus et al., 1986). The above plant viruses are transmitted by insect vectors in a semi-persistent manner. Unlike viruses that are transmitted in a circulative or persistent manner, semi-persistent viruses do not circulate through the insect’s body, which is more conducive to their spread in a short time (Chen et al., 2011). Previous studies have primarily focused on WFT transmission of MCMV. In our study, we investigated key transmission characteristics of MCMV by WFT, including acquisition access period (AAP), retention time within WFT, inoculation access period (IAP), and transmission efficiency. Further evidence supporting semi-persistent transmission will be supplemented to exclude the invasion of MCMV into salivary glands and the replication of the virus in the WFT, using the immunofluorescence experiment.

MCMV could potentially affect gene expression and the accumulation of transcripts in the thrips vector. In the present study, a high Q30 value indicates a higher data quality, with Q30 values ≥ 95.40%, enhancing the accuracy and reliability of downstream analysis such as gene expression analysis. We selected significant upregulated DEGs putatively associated with chemoreception, nutritional metabolism, immune response, and chitin-binding in the adults and larvae of thrips. Cathepsins, which play important roles in the adaptation of insects to plant secondary volatiles, are upregulated, as with previous studies (Terra et al., 2018; Tianzi et al., 2018). Chemoreception also plays a crucial role in regulating the life cycle of insects, as it is indispensable for insect odor recognition, food discrimination, and hosts selection (Leal, 2013; Sokolinskaya et al., 2020). Previous studies showed that OBPs were involved in the preference of insect vectors for virus-infected plants (Hu et al., 2019; He et al., 2023). Our transcriptome results also showed that *FoccOBP4* was upregulated in the viruliferous larvae of WFT, while *FoccGR14* and *FoccGR88* were upregulated in the viruliferous adults of WFT. This result indicated that chemosensory genes are involved in the preference of WFT for MCMV-infected maize plants. Semi-persistent virus achieves transmission by inducing behavioral changes in insect vectors. GABAR-mediated behavioral regulation may play critical role in the insect nervous system. Our transcriptome data showed that GABAR is upregulated in the viruliferous adults of WFT (**Figure 3F**). The foregut and maxillary stylets are retention regions of semi-persistent viruses; their surfaces are covered with cuticle consisting of a chitinous intima embedded in a protein matrix (Uzest et al., 2010). Therefore, cuticular proteins may function as receptors (Ng and Zhou, 2015). Our results confirmed that chitin-binding genes were upregulated in WFT larvae infected with MCMV (**Supplementary Table S5**), but downregulated in WFT larvae infected with persistent TSWV (Schneweis et al., 2017); this provides additional evidence to determine that WFT can transmit MCMV in a semi-persistent manner.

This study indicated that MCMV could alter the selection preferences of WFTs. Non-viruliferous WFTs prefer to settle on the MCMV-infected maize plants, while viruliferous WFTs prefer healthy maize plants, which could contribute to efficient viral transmission of MCMV. Our results are similar to previous studies, which showed that non-viruliferous *Rhopalosiphum padi* was more attracted by wheat plants infected with barley yellow dwarf virus (BYDV) than healthy plants, and the preference of viruliferous *R. padi* was significantly shifted to non-infected wheat plants (Jiménez-Martínez et al., 2004). *F. williamsi* and *Thrips tabaci* were significantly attracted to volatiles from MCMV-infected maize plants compared to healthy plants (Mwando et al., 2018). For the non-circulative plant viruses, virus-infected plants attract insect vectors through increased volatile emissions, and once the vectors have acquired the virus, they are deterred away from the infected plants to increase the transmission of virus (Blanc and Michalakis, 2016; Eigenbrode et al., 2018). WFT showed a higher preference for *N. benthamiana* plants transiently expressing MCMV CP, which is consistent with the findings observed in MCMV-infected maize. This result induces the releases of specific chemical signals from MCMV-infected plants that attract WFTs.

Through metabolomic profiling, MCMV induces changes in volatile profiles of maize plants, and 22 volatiles including nine terpenoids were upregulated in the MCMV-infected maize plants. Terpenoid is the largest group of volatiles after the plants suffering an external stimulus and primarily synthesized as secondary volatiles in plants, which could attract insects (Tholl, 2015; Wu et al., 2019; Huang et al., 2023). It is reported that guaiene as a kind of terpenoid is a potent oviposition attractant for both *Helicoverpa assulta* and *H. armigera* (Mo et al., 2023). β-Myrcene as a kind of monoterpene, which is also significantly attractive to *Helicoverpa armigera*, *Dioryctria pryeri*, and WFT (Rembold et al., 1991; Jin et al., 2023). In the previous studies, the concentration range of the Y-tube bioassay was typically ranged up to 100 μg/μL, and the remaining concentration was diluted by three to four gradients (Wang et al., 2022). In our study, the concentration range was from 10^−3^ μg/μL to 10 μg/μL, all within the detection threshold. Behavioral responses of WFT to volatiles are concentration dependent, and there are some differences in behavioral responses to volatiles at different concentrations (Ren et al., 2020). Lower concentrations of volatiles tend to attract insects, and higher concentrations tend to avoid them. For example, WFT more attracted to β-caryophyllene at concentrations of 0.1% and 0.001% compared to concentrations of 1% (Avellaneda et al., 2021). Our results also showed that the behavioral responses of WFT to MCMV-induced volatiles were concentration dependent. Among the chosen six upregulated volatiles, β-myrcene was significantly attractive to WFTs through the Y-tube olfactometer bioassay. In addition, an increase in β-myrcene production was detected in *N. benthamiana* plants transiently expressing MCMV CP. Despite the relatively low concentration of β-myrcene, it can still attract WFT effectively. β-myrcene is synthesized through the methylerythritol 4-phosphate (MEP) metabolic pathways with isopentenyl diphosphate (IPP) and dimethylallyl diphosphate (DMAPP) serving as substrates (Ye et al., 2020). Previous studies showed that β-myrcene was often produced in large quantities in plants after being attacked by pests, which may alter the behavior preference of insects (Rodriguez et al., 2011; Zhang et al., 2022). Current evidence has shown that viral infection can regulate the synthesis of plant volatiles (Li et al., 2014). For example, after plants were infected with banana bunchy top virus (BBTV), the quantities of some volatiles such as nonanal and β-myrcene, significantly increased, which may mediate the preference behavior of aphids toward infected bananas (Safari Murhububa et al., 2021). Another typical example is the research that when plants are infected with tomato yellow leaf curl virus (TYLCV), the viral genetic factor βC1 disrupts the activation process of terpene synthase genes mediated by the transcription factor MYC2, resulting in reduced resistance of infected plants against whiteflies (Li et al., 2014).

Our study demonstrates that β-myrcene is produced in maize after being infected with MCMV, which is verified in MCMV-infected *N. benthamiana* plants, and β-myrcene has a significant attractant effect on WFTs. Elicitors associated with the signal pathway of terpenoid biosynthesis, such as reactive oxygen species, jasmonic acid, and ethylene, could transactivate the transcription factors, which play essential function in regulating the expression of monoterpene synthesis genes, displaying critical application value for cultivation of transgenic plants (Huang et al., 2023). These studies provide theoretical significance for the synthesis of efficient attractants for the control of WFT in the future.

## 5 Conclusions

Our study provided important evidence supporting the semi-persistent transmission of MCMV by WFT, where the virus is retained by the insect vector for a brief period, which was obtained through bioassays and transcriptomic analysis. Furthermore, we showed for the first time that MCMV-induced β-myrcene attracting insect vectors maybe an important transmission mechanism of semi-persistent virus. This work will promote the research on the mechanisms of WFT–MCMV–maize interactions and provide a new insight for the combined control of MCMV and WFT through manipulation of plant volatiles and insect genes. With the development of insect molecular biology and gene editing technology, the application of genetically modified insects, synthetic attractants, and bait plants will become a reality in integrated pest management strategies.

## Data Availability

Transcriptome data is available via National Center for Biotechnology Information (NCBI) with bioProject accession number of PRJNA1050993. Raw reads have been deposited in the Sequence Read Archive (SRA) repository with the accession number of SRR27535893-SRR27535904. Metabolome data is available via China National GeneBank DataBase with the accession number of METM0000166 (https://db.cngb.org/mycngbdb/submissions/metabolism).
