# Supplementary material for: MCMV-infected maize attracts its insect vector Frankliniella occidentalis by inducing β-myrcene
Source: Front Plant Sci. 2024 Aug 21;15:1404271. doi: 10.3389/fpls.2024.1404271 (PMC11371577; doi:10.3389/fpls.2024.1404271)
Supplement: Supplementary file 1 [file Table1.docx]

**Supplementary material for**

**MCMV-infected maize attracts its insect vector *Frankliniella occidentalis* by inducing** **β-myrcene**

**Weiling Huang^1, 2^, Shujun Wei ^3^,** **Tao Zhou^1, 2^, Zaifeng Fan^1, 2^, Lijun Cao ^3^, Zhihong Li^1, 2^, Shaokun Guo^1, 2, *^**

1. Department of Plant Biosecurity, College of Plant Protection, China Agricultural University, Beijing, China

2. Key Laboratory of Surveillance and Management for Plant Quarantine Pests of Ministry of Agriculture and Rural Affairs, Beijing, China

3. Institute of Plant Protection, Beijing Academy of Agriculture and Forestry Sciences, Beijing, China

*** Correspondence:**

Shaokun Guo

[guoshaokun@cau.edu.cn](mailto:guoshaokun@cau.edu.cn)

**Table S1**. Primers used for RT-qPCR.

| **Primer name** | **Sequence (5'-3')** | **Application** |
| --- | --- | --- |
| CP-F | CAGGACGATTCATTGTGGCTAT | RT-qPCR |
| CP-R | CACGGTAGGACACGGAGTA | RT-qPCR |
| ACT-F | GGTATCGTCCTGGACTCTGGTG | RT-qPCR |
| ACT-R | GGGAAGGGCGTAACCTTCA | RT-qPCR |
| FPGS-F | ATCTCGTTGGGGATGTCTTG | RT-qPCR |
| FPGS-R | AGCACCGTTCAAATGTCTCC | RT-qPCR |

CP: coat protein gene of MCMV; ACT: the keeping house gene of *Frankliniella occidentalis*, actin; FPGS: the keeping house gene of maize.

Table S2 Detection of MCMV infection on maize leaves by RT-qPCR

| WFT | Days of post-acquisition / D | Ct Mean of Genes | | | | | | | | | |
| --- | --- | --- | --- | --- | --- | --- | --- | --- | --- | --- | --- |
|  |  | Replicate 1 | | Replicate 2 | | Replicate 3 | | Replicate 4 | | Replicate 5 | |
|  |  | *FPGS* | *CP* | *FPGS* | *CP* | *FPGS* | *CP* | *FPGS* | *CP* | *FPGS* | *CP* |
| A | 1 | 26.76 | 27.16 | 25.55 | 30.61 | 25.58 | 27.82 | 26.42 | 27.65 | 25.55 | 30.61 |
|  | 2 | 24.56 | Undetermined | 29.55 | Undetermined | 26.01 | 33.18 | 25.86 | Undetermined | 26.37 | Undetermined |
|  | 3 | 28.36 | Undetermined | 26.05 | Undetermined | 27.33 | Undetermined | 24.59 | Undetermined | 24.82 | Undetermined |
|  | 4 | 29.26 | Undetermined | 25.96 | Undetermined | 28.54 | Undetermined | 26.51 | Undetermined | 32.24 | Undetermined |
|  | 5 | 24.61 | Undetermined | 24.78 | Undetermined | 28.25 | Undetermined | 26.86 | Undetermined | 25.66 | Undetermined |
|  | 6 | 27.07 | Undetermined | 24.99 | Undetermined | 24.82 | Undetermined | 31.43 | Undetermined | 31.90 | Undetermined |
|  | 7 | 24.82 | Undetermined | 25.71 | Undetermined | 33.51 | Undetermined | 23.13 | Undetermined | 23.77 | Undetermined |
|  | 8 | 24.42 | Undetermined | 23.61 | Undetermined | 26.48 | Undetermined | 25.56 | Undetermined | 28.44 | Undetermined |
|  | 9 | 25.46 | Undetermined | 31.01 | Undetermined | 33.50 | Undetermined | 23.49 | Undetermined | 28.72 | Undetermined |
|  | 10 | 29.31 | Undetermined | / | / | 26.12 | Undetermined | / | / | / | / |
|  | 11 | 28.92 | Undetermined | / | / | 26.12 | Undetermined | / | / | / | / |
|  | 12 | 28.64 | Undetermined | / | / | 25.78 | Undetermined | / | / | / | / |
| L2 | 1 | 25.68 | 28.93 | 25.34 | 27.45 | 26.76 | 30.23 | 26.89 | 30.12 | 25.55 | 28.35 |
|  | 2 | 30.70 | Undetermined | 30.62 | Undetermined | 29.08 | Undetermined | 27.07 | 34.10 | 25.42 | Undetermined |
|  | 3 | 28.57 | Undetermined | 27.12 | Undetermined | 25.57 | Undetermined | 24.77 | Undetermined | 25.75 | Undetermined |
|  | 4 | 25.16 | 37.57 | 25.32 | 38.40 | 24.22 | 38.50 | 24.96 | Undetermined | 24.00 | 38.22 |
|  | 5 | 28.35 | 38.97 | 28.09 | Undetermined | 33.61 | 38.35 | 28.12 | 38.72 | 32.25 | Undetermined |
|  | 6 | 28.45 | 38.97 | 32.14 | Undetermined | 36.68 | 38.35 | 28.76 | 38.72 | 34.61 | Undetermined |
|  | 7 | 34.28 | Undetermined | 24.70 | Undetermined | Undetermined | Undetermined | 24.10 | 39.55 | 24.70 | 37.68 |
|  | 8 | 24.28 | Undetermined | 24.48 | Undetermined | 27.98 | Undetermined | 24.21 | Undetermined | 26.77 | Undetermined |
|  | 9 | 28.12 | Undetermined | 29.14 | Undetermined | 30.14 | Undetermined | 24.81 | Undetermined | 27.74 | Undetermined |
|  | 10 | 25.52 | Undetermined | 31.69 | Undetermined | 24.90 | Undetermined | 26.86 | Undetermined | 24.34 | Undetermined |
|  | 11 | 24.48 | Undetermined | / | / | 27.98 | Undetermined | 24.21 | Undetermined | / | / |
|  | 12 | 28.65 | Undetermined | / | / | 24.48 | Undetermined | 25.65 | Undetermined | / | / |

A: adults of WFT. L2: the second instar larvae of WFT. Undetermined: no MCMV was detected. /: all WFTs in this replicate died, and no maize leaves were collected.

Table S3 Summary of Illumina sequencing data

| **Sample** | **Raw_reads** | **Raw_bases** | **Clean_reads** | **Clean_Bases** | **Error%** | **Q20%** | **Q30%** | **GC%** |
| --- | --- | --- | --- | --- | --- | --- | --- | --- |
| AN1 | 49952996 | 7542902396 | 49574092 | 7383718727 | 0.0236 | 98.57 | 95.58 | 46.91 |
| AN2 | 49058696 | 7407863096 | 48732722 | 7312628896 | 0.0236 | 98.61 | 95.55 | 47.17 |
| AN3 | 53874036 | 8134979436 | 53549654 | 8038116307 | 0.0232 | 98.75 | 95.97 | 47.13 |
| AV1 | 56143804 | 8477714404 | 55779812 | 8348625300 | 0.0235 | 98.64 | 95.67 | 47.91 |
| AV2 | 49702568 | 7505087768 | 49423754 | 7426517979 | 0.0234 | 98.68 | 95.72 | 47.15 |
| AV3 | 64322358 | 9712676058 | 63930978 | 9563065335 | 0.0232 | 98.75 | 95.97 | 48.29 |
| LN1 | 49070116 | 7409587516 | 48742896 | 7286775490 | 0.0236 | 98.59 | 95.62 | 46.92 |
| LN2 | 50778738 | 7667589438 | 50461906 | 7571875583 | 0.0236 | 98.62 | 95.63 | 48.17 |
| LN3 | 59091300 | 8922786300 | 58660716 | 8787333916 | 0.0237 | 98.56 | 95.5 | 47.78 |
| LV1 | 53323708 | 8051879908 | 52965974 | 7927372755 | 0.0237 | 98.54 | 95.45 | 47.13 |
| LV2 | 47252306 | 7135098206 | 46961020 | 7034645724 | 0.0234 | 98.67 | 95.8 | 46.68 |
| LV3 | 52506694 | 7928510794 | 52139946 | 7829550022 | 0.0238 | 98.51 | 95.4 | 48.16 |

AN: WFT adult feeding on healthy maize; AV: WFT adult feeding on MCMV-infected maize; LN: WFT larvae feeding on healthy maize; LV: WFT larvae feeding on MCMV-infected maize.

Table S4 the statistics of RNA-seq reads align to genome.

| **Sample** | **Total_reads** | **Total_mapped** | **Multiple_mapped** | **Unique_mapped** |
| --- | --- | --- | --- | --- |
| AN1 | 49574092 | 43050569(86.84%) | 908894(1.83%) | 42141675(85.01%) |
| AN2 | 48732722 | 41882686(85.94%) | 834026(1.71%) | 41048660(84.23%) |
| AN3 | 53549654 | 45477554(84.93%) | 947941(1.77%) | 44529613(83.16%) |
| AV1 | 55779812 | 47457282(85.08%) | 1120955(2.01%) | 46336327(83.07%) |
| AV2 | 49423754 | 42734531(86.47%) | 914803(1.85%) | 41819728(84.61%) |
| AV3 | 63930978 | 54989564(86.01%) | 1236766(1.93%) | 53752798(84.08%) |
| LN1 | 48742896 | 41170788(84.47%) | 832310(1.71%) | 40338478(82.76%) |
| LN2 | 50461906 | 43408940(86.02%) | 854113(1.69%) | 42554827(84.33%) |
| LN3 | 58660716 | 49978643(85.2%) | 1041369(1.78%) | 48937274(83.42%) |
| LV1 | 52965974 | 44982267(84.93%) | 879129(1.66%) | 44103138(83.27%) |
| LV2 | 46961020 | 39745274(84.63%) | 736547(1.57%) | 39008727(83.07%) |
| LV3 | 52139946 | 44237884(84.84%) | 931961(1.79%) | 43305923(83.06%) |

AN: WFT adult feeding on healthy maize; AV: WFT adult feeding on MCMV-infected maize; LN: WFT larvae feeding on healthy maize; LV: WFT larvae feeding on MCMV-infected maize.

Table S5 The chitin binding related genes and tubulin-related genes for adult and larvae of WFT after TSWV or MCMV acquisition

| **Gene_ID** | **Gene_name** | **WFT samples** | **Direction^1^** | **Fold_change** | **Description** | **Reference** |
| --- | --- | --- | --- | --- | --- | --- |
| gene-FOCC_FOCC001394 | FOCC_FOCC001394 | A-M/CK | UP | 2.29 | chitin binding | This study |
| gene-FOCC_FOCC013088 | FOCC_FOCC013088 | A-M/CK | UP | 1.98 | chitin binding | This study |
| gene-FOCC_FOCC006559 | FOCC_FOCC006559 | L-M/CK | UP | 2.27 | ------ | This study |
| gene-FOCC_FOCC014694 | FOCC_FOCC014694 | L-M/CK | UP | 2.11 | ------ | This study |
| XLOC_003434 | - | L-T/CK | DOWN | 2.21 | chitin-based cuticle development | Schneweis et al., 2017 |
| FOCC016851 | FoccTmpM016851 | L-T/CK | DOWN | 1.88 | chitin-based cuticle development | Schneweis et al., 2017 |
| FOCC006492 | FoccTmpM006492 | L-T/CK | DOWN | 2.24 | chitin-based cuticle development | Schneweis et al., 2017 |
| FOCC015389 | FoccTmpM015389 | L-T/CK | DOWN | 1.98 | chitin-based cuticle development | Schneweis et al., 2017 |
| FOCC008596 | Dmel-Tmp-cpr62bc | L-T/CK | DOWN | 1.48 | chitin-based cuticle development | Schneweis et al., 2017 |
| FOCC006495 | FoccTmpM006495 | L-T/CK | DOWN | 1.89 | chitin-based cuticle development | Schneweis et al., 2017 |
| FOCC006022 | FoccTmpM006022 | L-T/CK | DOWN | 1.84 | chitin-based cuticle development | Schneweis et al., 2017 |
| FOCC009240 | FoccTmpS009240 | L-T/CK | DOWN | 1.95 | chitin binding; chitin metabolic process | Schneweis et al., 2017 |
| FOCC017516 | FoccTmpM017516 | L-T/CK | DOWN | 1.57 | chitin binding; chitin metabolic process | Schneweis et al., 2017 |
| CUFF | Dmel-Tmp-pu | A-T/CK | UP | 1.52 | chitin-based cuticle sclerotization | Schneweis et al., 2017 |
| CUFF | Dmel-Tmp-cht3 | A-T/CK | UP | 4.79 | chitin binding; chitin catabolic process | Schneweis et al., 2017 |

A-M: adult-MCMV. L-M: larvae-MCMV. L-T: larvae-TSWV. A-T: adult-TSWV.

^1^Direction of WFT samples (B/C): UP and DOWN indicate high and low expression level of B.

Table S6 The immune response, nutritional metabolism and chemosensory-related genes for adult and larvae of WFT after MCMV acquisition

|  | **Gene_ID** | **Gene_name** | **V1** | **V2** | **V3** | **N1** | **N2** | **N3** | **Fold_change** | **Pvalue** | **Padjust** |
| --- | --- | --- | --- | --- | --- | --- | --- | --- | --- | --- | --- |
| Adult | gene-FOCC_FOCC001394 | FoccPeritrophin-1-like | 276.17 | 229.72 | 231.88 | 123.81 | 101.78 | 96.45 | 2.863083 | 9.50E-10 | 7.54E-08 |
|  | gene-FOCC_FOCC004423 | FoccGR88 | 3275.37 | 2685.8 | 2804.2 | 1092.01 | 917.16 | 1089.67 | 3.079338 | 4.52E-13 | 4.90E-11 |
|  | gene-FOCC_FOCC005700 | FoccGR14 | 563.07 | 215.81 | 434.78 | 109.38 | 116.14 | 157.64 | 4.400203 | 0.000446 | 0.014823 |
|  | gene-FOCC_FOCC006309 | FoccPGRP-LB | 8.56 | 11.12 | 11.38 | 5.48 | 7.36 | 5.26 | 2.035937 | 1.34E-07 | 8.70E-06 |
|  | gene-FOCC_FOCC006989 | FoccSOCS-5 | 32.94 | 27.2 | 33.18 | 11.93 | 9.43 | 3.35 | 4.21715 | 9.47E-08 | 6.34E-06 |
|  | gene-FOCC_FOCC009124 | FoccCBP1 | 1201.76 | 1044.46 | 1077.6 | 283.39 | 337.42 | 321.14 | 4.294209 | 1.21E-23 | 2.64E-21 |
|  | gene-FOCC_FOCC009159 | FoccACE | 182.23 | 148.99 | 183.02 | 54.3 | 73.25 | 80.81 | 2.974563 | 2.72E-10 | 2.24E-08 |
|  | gene-FOCC_FOCC009474 | FoccCLP1 | 55.21 | 65.05 | 50.45 | 3.93 | 4.32 | 1.45 | 21.03083 | 5.07E-48 | 3.10E-45 |
|  | gene-FOCC_FOCC013475 | FoccVLDLR | 595.94 | 581.95 | 529.7 | 313.82 | 336.64 | 344.95 | 2.06761 | 4.89E-08 | 3.38E-06 |
|  | gene-FOCC_FOCC013541 | FoccHex | 0.89 | 1.75 | 2.77 | 0.16 | 0.89 | 0.57 | 3.785412 | 0.001648 | 0.044119 |
|  | gene-FOCC_FOCC017964 | FoccGABAR | 3.22 | 3.78 | 4.08 | 1.93 | 1.99 | 0.68 | 2.897063 | 0.000196 | 0.007152 |
| Larvae | gene-FOCC_FOCC014404 | FoccSpz | 17.73 | 14.75 | 15.62 | 34.58 | 36.84 | 26.07 | 0.489671099 | 2.12E-13 | 9.47E-11 |
|  | gene-FOCC_FOCC006309 | FoccPGRP-LB | 12.63 | 16.69 | 13.35 | 6.5 | 8.27 | 5.85 | 2.044370087 | 8.69E-08 | 1.26E-05 |
|  | gene-FOCC_FOCC006559 | FoccPeritrophin-A-like | 57.07 | 68.2 | 59.88 | 24.52 | 29.26 | 27.64 | 2.236920023 | 5.83E-18 | 5.40E-15 |
|  | gene-FOCC_FOCC003738 | FoccTrypsin7a | 40.09 | 53.52 | 42.36 | 9.21 | 9.28 | 9.02 | 4.803637384 | 1.40E-26 | 2.41E-23 |
|  | gene-FOCC_FOCC016401 | FoccCHYA-like | 2.9 | 5.94 | 2.78 | 0 | 0.9 | 0.86 | 6.28476069 | 0.000336748 | 0.015729284 |
|  | gene-FOCC_FOCC002858 | FoccOBP4 | 11.57 | 2.41 | 8.73 | 1.45 | 0.29 | 0.25 | 11.0380106 | 0.00032033 | 0.015138424 |

Table S7 Up-regulated and down-regulated metabolites in MCMV-infected maize plants

| **Compounds** | **V-1** | **V-2** | **V-3** | **V-4** | **V-5** | **V-6** | **N-1** | **N-2** | **N-3** | **N-4** | **N-5** | **N-6** |
| --- | --- | --- | --- | --- | --- | --- | --- | --- | --- | --- | --- | --- |
| 2,4-Dimethyldecane | -1.20 | -0.95 | -0.80 | -0.62 | -0.42 | -1.00 | 0.41 | 1.98 | -0.02 | 1.14 | 0.77 | 0.69 |
| 2-Isopropyl-5-methylhex-2-enal | -1.83 | -0.93 | -0.62 | -0.74 | -0.16 | -0.49 | 0.09 | 1.63 | 0.11 | 1.11 | 0.90 | 0.93 |
| 2-Methylbutyl 3-methylbutanoate | -1.87 | -0.74 | -0.76 | -0.61 | -0.11 | -0.52 | 0.16 | 2.02 | 0.26 | 0.62 | 0.71 | 0.83 |
| (E,E)-2,4-Undecadienal | -1.33 | -0.73 | -0.19 | -0.63 | -0.90 | -0.99 | 1.40 | 1.36 | -0.51 | 1.18 | 0.45 | 0.89 |
| Dimethyl phthalate | -0.82 | -0.48 | -0.76 | -0.90 | -1.08 | -0.97 | 0.07 | 1.07 | 0.10 | 0.84 | 1.93 | 1.01 |
| 2,4-Dimethyl-2-oxazoline-4-methanol | -0.52 | -0.44 | -0.93 | -0.70 | -0.65 | -0.63 | -0.59 | 1.05 | -0.62 | 1.88 | 1.66 | 0.49 |
| 4,8-Dimethyl-1,7-nonadiene | -0.74 | -0.44 | -0.90 | -0.53 | -0.87 | -0.78 | -0.04 | 1.02 | -0.53 | 1.97 | 1.59 | 0.26 |
| 3,5-Dimethylcyclohexanol | -1.05 | -0.81 | -1.02 | -0.55 | -0.57 | -0.97 | 0.89 | 1.37 | 1.62 | 0.79 | 0.73 | -0.42 |
| Dihydro-2-methyl-3(2H)-furanone | 0.23 | -1.07 | -1.26 | -0.29 | -1.15 | -0.70 | 1.38 | 0.63 | 1.96 | -0.03 | -0.14 | 0.45 |
| Octyl formate | 0.65 | -0.94 | -0.81 | -0.79 | -0.82 | -0.80 | 0.16 | 2.52 | 0.55 | 0.49 | 0.20 | -0.40 |
| Heptyl acetate | 0.54 | -0.87 | -0.81 | -0.76 | -0.80 | -0.86 | 0.15 | 2.58 | 0.42 | 0.55 | 0.20 | -0.35 |
| cis-6-Nonenal | 0.30 | -0.93 | -0.84 | -0.97 | -0.99 | -0.81 | 0.55 | 2.37 | 0.63 | 0.54 | 0.32 | -0.17 |
| 2-Nonanol | 0.47 | -0.91 | -0.84 | -0.82 | -0.88 | -0.90 | 0.47 | 2.49 | 0.43 | 0.51 | 0.21 | -0.24 |
| 1,2,3,5,6,7-Hexahydro-1,1,2,3,3-pentamethyl-4H-inden-4-one | 2.33 | 0.23 | 0.63 | 0.49 | 0.98 | -0.14 | -0.55 | -0.77 | -0.78 | -0.28 | -1.01 | -1.13 |
| Geranyl butyrate | 0.50 | 1.17 | 0.39 | 2.41 | 0.17 | -0.33 | -0.61 | -0.94 | -0.70 | -0.43 | -0.81 | -0.81 |
| 2,3-Dehydro-1,8-cineole | -0.08 | 1.11 | 0.40 | 0.35 | 1.24 | 1.90 | -0.65 | -0.96 | -1.19 | -0.47 | -0.84 | -0.82 |
| 6-Methylhept-5-en-2-ol | -0.40 | 0.66 | 0.13 | 0.57 | 1.52 | 2.01 | -0.51 | -0.92 | -1.16 | -0.43 | -0.72 | -0.74 |
| 2-Pentylfuran | 0.01 | 0.68 | 0.39 | 0.52 | 1.77 | 1.62 | -0.79 | -0.68 | -1.19 | -0.54 | -0.96 | -0.83 |
| beta-Myrcene | 0.67 | 0.74 | 0.17 | 0.88 | 1.03 | 1.76 | -0.53 | -0.98 | -1.25 | -0.51 | -0.96 | -1.03 |
| 2,6-Dimethyl-2-trans-6-octadiene | 0.28 | 1.24 | -0.11 | 0.45 | 0.41 | 2.32 | -0.74 | -0.89 | -0.81 | -0.49 | -0.81 | -0.86 |
| (3S,3aR,3bR,4S,7R,7aR)-4-Isopropyl-3,7-dimethyloctahydro-1H-cyclopenta[1,3]cyclopropa[1,2]benzen-3-ol | 0.71 | 0.79 | -0.03 | 0.26 | 0.51 | 2.30 | -1.05 | -0.92 | -1.09 | -0.08 | -0.49 | -0.92 |
| cis-beta-Copaene | 0.49 | 2.07 | 1.38 | 0.14 | 0.32 | 0.17 | -0.99 | -1.03 | -1.29 | -0.07 | -0.64 | -0.55 |
| Resorcinol monoacetate | 0.26 | 1.40 | 1.33 | 0.90 | 0.25 | 0.84 | -1.40 | -1.20 | -1.29 | -0.07 | -0.36 | -0.67 |
| Methyl p-tolyloxyacetate | 0.15 | 1.64 | 1.17 | 0.72 | 0.26 | 0.97 | -1.36 | -1.19 | -1.28 | -0.16 | -0.37 | -0.57 |
| (-)-alpha-Alaskene | -0.36 | 2.21 | 0.81 | 0.27 | -0.15 | 0.74 | -1.44 | -0.89 | -1.26 | 0.32 | 0.11 | -0.36 |
| 7-Octylidenebicyclo[4.1.0]heptane | -0.31 | 2.06 | 0.60 | 0.45 | -0.03 | 0.83 | -1.46 | -1.15 | -1.31 | 0.38 | 0.20 | -0.26 |
| alpha-Farnesene | -0.23 | 2.00 | 0.74 | 0.31 | 0.00 | 0.89 | -1.47 | -0.99 | -1.35 | 0.60 | -0.08 | -0.43 |
| N-Butyl-1-(pyridin-2-yl)methanimine | -0.45 | 2.29 | 1.06 | -0.02 | 0.03 | 0.52 | -1.40 | -0.82 | -1.15 | 0.26 | 0.03 | -0.34 |
| 5-Ethyl-2(5H)-furanone | -0.74 | 1.55 | 1.74 | -0.24 | 1.27 | 0.12 | -0.95 | -0.78 | -1.12 | 0.20 | -0.57 | -0.48 |
| Cyclohexanecarbaldehyde | -0.78 | 1.90 | 0.57 | 0.43 | 1.34 | 0.53 | -1.08 | -0.91 | -1.27 | 0.28 | -0.47 | -0.54 |
| 3-Ethylbenzaldehyde | -0.48 | 2.51 | 1.07 | -0.30 | 0.31 | 0.17 | -0.94 | -0.43 | -1.21 | 0.27 | -0.37 | -0.61 |
| Di-epi-alpha-cedrene-(I) | 0.07 | 1.29 | 0.86 | 0.46 | -0.02 | 1.79 | -1.31 | -1.19 | -1.28 | 0.10 | -0.17 | -0.60 |
| Butyl benzoate | 0.00 | 1.07 | 0.94 | 0.77 | 0.23 | 1.65 | -1.33 | -1.24 | -1.32 | 0.15 | -0.24 | -0.68 |
| beta-Himachalene | -0.49 | 1.44 | 0.68 | 0.00 | -0.06 | 2.00 | -1.23 | -1.00 | -1.07 | 0.48 | -0.21 | -0.54 |
| Linalyl butyrate | 0.43 | 1.15 | 0.90 | -0.07 | 0.31 | 1.31 | -1.68 | -1.19 | -1.54 | 0.29 | 0.29 | -0.20 |

N: healthy maize plants; V: MCMV-infected maize plants.
